# Physiological discordance between clinical examination, bioimpedance spectroscopy, and lung ultrasound in maintenance hemodialysis patients without heart failure

**DOI:** 10.3389/fneph.2026.2000700

**Published:** 2026-10-09

**Authors:** Irwing R. Benites-Flores, Javier Rodríguez Revilla, Alvaro Taype-Rondan, Liliana C. Fernández-Anticona, Pedro Augusto Prado Ramírez, Nelly Seminario Alegre, Celia Rodríguez Tudero, Esperanza Moral Berrio, Elena Jimenez Mayor, Avinash Chandu Nanwani, José C. De la Flor

**Affiliations:** 1 Department of Nephrology, Hospital Alberto Barton Thompson, Callao, Peru; 2 Department of Nephrology, Hospital Edgardo Rebagliati Martins, Lima, Peru; 3 Unidad de Investigación para la Generación y Síntesis de Evidencias en Salud, Vicerrectorado de Investigación, Universidad San Ignacio de Loyola, Lima, Peru; 4 EVITA Research Center, EviSalud – Evidencias en Salud, Lima, Peru; 5 Department of Nephrology, Hospital Universitario de Salamanca, Salamanca, Spain; 6 Department of Surgery, Faculty of Medicine, University of Salamanca, Salamanca, Spain; 7 Department of Nephrology, Hospital General Universitario de Ciudad Real, Ciudad Real, Spain; 8 Department of Nephrology, Hospital Santa Bárbara, Soria, Spain; 9 Department of Nephrology, Hospital General de Fuerteventura, Fuerteventura, Spain; 10 Department of Nephrology, Hospital Central de la Defensa Gómez Ulla, Madrid, Spain; 11 Department of Medicine and Medical Specialties, Faculty of Medicine and Health Sciences, University of Alcalá, Madrid, Spain

**Keywords:** bioimpedance spectroscopy, hemodialysis, lung ultrasound, point-of-care ultrasonography, pulmonary congestion, volume assessment

## Abstract

**Background and objectives:**

Accurate volume assessment in maintenance hemodialysis remains challenging because no single bedside method fully characterizes extracellular fluid overload. We evaluated discordance among clinical examination, bioimpedance spectroscopy, and abbreviated six-zone lung ultrasound, the prevalence of subclinical pulmonary congestion, and factors associated with pulmonary congestion.

**Methods:**

In this single-center cross-sectional study, 116 adults receiving maintenance hemodialysis without clinically recognized heart failure underwent standardized clinical examination, whole-body bioimpedance spectroscopy, and predialysis six-zone lung ultrasound. Subclinical pulmonary congestion was defined as a prespecified total B-line count of ≥3 in clinically euvolemic patients. Agreement was assessed using Cohen’s kappa, and independent associations were evaluated using multivariable Poisson regression.

**Results:**

Among 49 clinically euvolemic patients, 27 (55.1%) had subclinical pulmonary congestion, representing 23.3% of the cohort. Clinical examination showed fair agreement with lung ultrasound for ≥3 B-lines (κ = 0.28; 95% CI, 0.11–0.45), whereas agreement between lung ultrasound and bioimpedance-defined overhydration was slight (κ = 0.16; 95% CI, −0.03 to 0.35). Bioimpedance-derived overhydration, but not interdialytic weight gain, was independently associated with subclinical pulmonary congestion.

**Conclusion:**

Discordance among clinical examination, bioimpedance spectroscopy, and lung ultrasound was common and is consistent with the hypothesis that these modalities capture partially distinct aspects of volume status and pulmonary congestion. Abbreviated lung ultrasound frequently identified clinically occult pulmonary congestion despite apparent clinical euvolemia, supporting a multidimensional approach to bedside volume assessment in maintenance hemodialysis.

## Introduction

Volume overload is one of the most important modifiable cardiovascular risk factors in patients receiving maintenance hemodialysis and remains a major contributor to hospitalization, heart failure, and premature mortality (1). Consequently, accurate assessment of volume status is a cornerstone of dialysis care, guiding fluid management, ultrafiltration, and cardiovascular risk reduction. Defining true volume status, however, remains challenging because extracellular fluid overload is a heterogeneous physiological process that cannot be fully characterized by a single measurement. Importantly, volume overload is not synonymous with congestion. Whereas volume overload denotes increased extracellular fluid volume, congestion reflects fluid accumulation or redistribution within vascular and interstitial compartments, which does not necessarily parallel the degree or distribution of extracellular fluid expansion. In contemporary nephrology practice, clinicians integrate clinical examination, interdialytic weight gain, blood pressure, and objective tools such as bioimpedance spectroscopy (BIS). Although each approach provides valuable information, each captures distinct physiological domains of extracellular fluid overload and congestion (2–4).

Pulmonary congestion is an early manifestation of extracellular fluid redistribution and may precede overt signs of systemic fluid overload. Consequently, patients without peripheral edema, abnormal lung auscultation, or dyspnea may still have increased extravascular lung water, indicating subclinical pulmonary congestion that often goes undetected by conventional clinical assessment. Persistent pulmonary congestion is clinically important because it is consistently associated with hospitalization, cardiovascular events, and mortality in patients receiving maintenance hemodialysis (5–8).

Lung ultrasound (LUS) has emerged as a rapid, non-invasive bedside technique for detecting pulmonary congestion through B-line quantification (9). Landmark studies have shown that pulmonary congestion is highly prevalent in maintenance hemodialysis, often persists despite apparently adequate clinical assessment, and decreases after ultrafiltration, supporting LUS as a dynamic marker of lung fluid accumulation (5–8). More recently, abbreviated LUS protocols have demonstrated diagnostic performance comparable to the traditional 28-zone examination while substantially reducing acquisition time, making them feasible for routine use in dialysis units (10–12). Longitudinal studies further suggest that abbreviated LUS can safely support fluid management while enabling serial monitoring of pulmonary congestion (13).

Despite these advances, an important knowledge gap remains. Most previous studies have evaluated LUS as an isolated diagnostic or prognostic tool rather than as part of an integrated assessment of volume status. Clinical examination, blood pressure, BIS, and LUS assess volume status and congestion using different clinical and physiological measurements, and discordance among these methods has been reported (14–16). Whether this discordance reflects partially distinct physiological information or other methodological and patient-related factors remains uncertain. Characterizing these patterns of discordance may help clarify the relationships among bedside volume assessment methods in maintenance hemodialysis.

Therefore, this study aimed to characterize discordance among standardized clinical examination, BIS, and an abbreviated six-zone LUS protocol in maintenance hemodialysis patients without clinically recognized heart failure. Agreement between assessment methods was evaluated to quantify classification concordance, and the prevalence of subclinical pulmonary congestion and factors associated with pulmonary B-line accumulation were determined.

## Materials and methods

### Study design and setting

We conducted a single-center cross-sectional analytical study in the Department of Nephrology at Hospital Alberto Barton Thompson, a tertiary referral hospital of the Peruvian Social Health Insurance system (EsSalud) in Callao, Peru.

Consecutive eligible patients were enrolled and evaluated between November 2024 and July 2025. The study is reported according to the Strengthening the Reporting of Observational Studies in Epidemiology (STROBE) guidelines (17).

### Study population

Adult patients (≥18 years) receiving maintenance hemodialysis for at least three months were consecutively enrolled during the study period. Patients were excluded if they had a recent hospitalization for acute decompensated heart failure, active pulmonary infection, diffuse interstitial lung disease, or New York Heart Association (NYHA) class III and IV heart failure. Patients with previously established heart failure were excluded based on available cardiology records. Transthoracic echocardiography was available in a subgroup of patients as part of routine clinical care. Because echocardiographic examinations were not systematically performed for research purposes, echocardiographic availability was not considered an inclusion criterion. Instead, these examinations were used to further characterize cardiac structure and function and to support the absence of clinically recognized heart failure when available.

### Study procedures

All study procedures were performed immediately before the midweek hemodialysis session to ensure that all measurements reflected the same physiological state before ultrafiltration. On the study day, participants underwent standardized clinical examination, BIS, and LUS assessment. Routine laboratory data were obtained from the closest predialysis blood sample according to the dialysis unit protocol, and dialysis-related variables were extracted from the medical records.

### Standardized clinical examination

Demographic and dialysis-related characteristics, including age, sex, time on hemodialysis, vascular access, dialysis frequency, treatment duration, residual urine output, and relevant comorbidities were obtained from the medical records. A standardized clinical examination was performed in all participants and included assessment of dyspnea, pulmonary auscultation, peripheral edema, predialysis systolic and diastolic blood pressure, and interdialytic weight gain.

### Laboratory measurements

Routine laboratory variables were collected from the closest predialysis blood sample and included hemoglobin, serum albumin, sodium, potassium, calcium, phosphorus, ferritin, transferrin saturation, and other biochemical parameters routinely used in dialysis care.

### Bioimpedance spectroscopy

Whole-body BIS was performed before dialysis using the Body Composition Monitor (BCM^®^, Fresenius Medical Care, Bad Homburg, Germany) (18). Measurements were performed by dialysis nurses trained in bioimpedance assessment before the midweek hemodialysis session according to the manufacturer’s recommendations. Patients were examined in the supine position after removal of metallic objects, and surface electrodes were placed on the ipsilateral hand and foot. The BCM provides estimates of intracellular water, extracellular water, lean tissue mass, fat tissue mass, and the degree of overhydration using validated bioelectrical impedance models. The primary bioimpedance variable was overhydration (OH), expressed in liters, representing the volume of excess extracellular fluid relative to the patient’s normohydrated weight.

### Lung ultrasound assessment

LUS was performed as a point-of-care ultrasound (POCUS) examination immediately before the midweek hemodialysis session using standardized acquisition protocols, a predefined quality assurance process, and a six-zone scanning protocol selected for its practicality for routine implementation in dialysis units while maintaining adequate diagnostic performance. Examinations were performed using a Butterfly iQ+ handheld ultrasound system (Butterfly Network Inc., Guilford, CT, USA) equipped with a phased-array transducer. Patients were examined in a supine or near-supine position following a standardized six-zone protocol that included the second and fourth intercostal spaces along the midclavicular line and the fourth intercostal space along the midaxillary line in each hemithorax (10, 12).

Before B-line quantification, each examination included a systematic assessment of the pleural line and pleural space. Pleural line abnormalities, pleural effusion, and other relevant sonographic findings were documented whenever present.

A B-line was defined according to international recommendations as a discrete laser-like vertical hyperechoic reverberation artifact arising from the pleural line, extending to the bottom of the screen without fading, and moving synchronously with lung sliding (19). The total pulmonary B-line count was calculated as the sum of B-lines identified across the six lung zones.

Subclinical pulmonary congestion was operationally defined as a total B-line count of ≥3 in clinically euvolemic patients. This threshold was prespecified before examination of the study data and was not derived from the present dataset. It was selected as a low B-line threshold intended to identify clinically occult pulmonary fluid accumulation and characterize clinical–ultrasound discordance rather than to define clinically overt or moderate-to-severe pulmonary congestion. The threshold was informed by previous hemodialysis literature using B-line categories beginning at ≥3 (20). Because B-line thresholds vary across scanning protocols and no universally accepted cutoff exists for subclinical pulmonary congestion using an abbreviated six-zone examination, sensitivity analyses were additionally performed using thresholds of ≥5 and ≥10 B-lines.

LUS acquisition was independently performed by two nephrologists formally trained through the ASARUC Academy Multisystem POCUS I Program, who remained blinded to the results of the clinical examination, laboratory variables, bioimpedance measurements, dialysis-related variables, and echocardiographic findings.

All ultrasound images were digitally stored and subsequently underwent independent review by the same two nephrologists. Whenever disagreement existed regarding B-line quantification or additional sonographic findings, the final interpretation was established by consensus before statistical analysis.

### Echocardiographic assessment

Transthoracic echocardiographic data were available for a subgroup of participants as part of routine clinical practice. When available, left ventricular ejection fraction, diastolic function, tricuspid annular plane systolic excursion (TAPSE), significant valvular abnormalities, estimated pulmonary hypertension, and pericardial effusion were recorded. These variables were considered descriptive and exploratory and were not incorporated into the primary analyses because echocardiography was not uniformly available for all participants.

### Study definitions

Patients were classified as clinically congested when at least one predefined sign suggestive of systemic or pulmonary congestion was present during the standardized clinical examination (dyspnea, peripheral edema, or pulmonary crackles on auscultation). Clinical euvolemia was defined as the absence of all predefined signs (21).

Subclinical pulmonary congestion was defined as the presence of ≥3 B-lines among patients considered clinically euvolemic. This definition operationalizes the concept of clinical–ultrasound discordance, whereby standardized clinical examination indicates clinical euvolemia whereas LUS demonstrates objective pulmonary congestion (7, 8, 10).

To evaluate the robustness of our findings, sensitivity analyses were additionally performed using thresholds of ≥5 and ≥10 B-lines. Bioimpedance-defined overhydration was defined as BCM overhydration >1.1 L. Elevated interdialytic weight gain was defined as >4% of dry body weight (8, 10).

### Study outcomes

The primary outcome was pulmonary congestion quantified by the total B-line count obtained using the standardized six-zone LUS protocol.

Secondary outcomes included the prevalence of subclinical pulmonary congestion, the degree of agreement between standardized clinical examination, BIS, and LUS, the relationships among objective markers of volume status, and the identification of clinical and dialysis-related factors associated with total pulmonary B-line count.

### Statistical analysis

Statistical analyses were designed to characterize the multidimensional assessment of volume status by evaluating agreement, discordance, and associations among clinical examination, BIS, blood pressure, and LUS. Continuous variables were summarized as mean ± standard deviation or median with interquartile range, according to their distribution, whereas categorical variables were expressed as frequencies and percentages. Comparisons between groups were performed using Student’s t test or the Mann–Whitney U test for continuous variables and the chi-square or Fisher’s exact test for categorical variables, as appropriate.

Agreement between standardized clinical examination, BIS, and LUS was quantified using Cohen’s kappa coefficient (22). Associations between continuous variables were explored using Spearman’s rank correlation coefficient. Because the primary outcome was common and the study was cross-sectional, multivariable Poisson regression with robust variance was used to estimate adjusted prevalence ratios for subclinical pulmonary congestion among clinically euvolemic patients. Variables were selected based on clinical relevance and availability. Logistic regression and alternative B-line thresholds were used as sensitivity analyses. A two-sided P value <0.05 was considered statistically significant (23). Additional exploratory analyses evaluated the relationships between predialysis blood pressure, bioimpedance-derived overhydration, and total pulmonary B-line count to further characterize the relationships among these measures of volume status and pulmonary congestion.

All analyses were performed using R software (R Foundation for Statistical Computing, Vienna, Austria). All statistical tests were two-sided, and a *P* value <0.05 was considered statistically significant.

## Results

### Study population

A total of 116 patients receiving maintenance hemodialysis were included in the study illustrated in **Figure 1**. The median age was 67 years, 69 patients (59.5%) were male, and median time on hemodialysis was 37 months (IQR, 16–68). Median interdialytic weight gain was 2.0 kg, median BCM-derived overhydration was 2.5 L (IQR, 1.3–3.6), and median residual urine output was 300 mL/day (IQR, 200–500). The median pulmonary B-line count was 8 (IQR, 2–13). Clinical evidence of congestion was present in 67 patients (57.8%), whereas 49 (42.2%) had no clinical manifestations of congestion. Overall, 82 patients (70.7%) had pulmonary congestion defined as ≥3 B-lines, and 48 (41.4%) had moderate-to-severe pulmonary congestion (≥10 B-lines). Baseline characteristics are summarized in **Table 1**. The diagnostic performance of the prespecified B-line thresholds for identifying clinically overt congestion is summarized in **Supplementary Table S1**.

**Figure 1:**
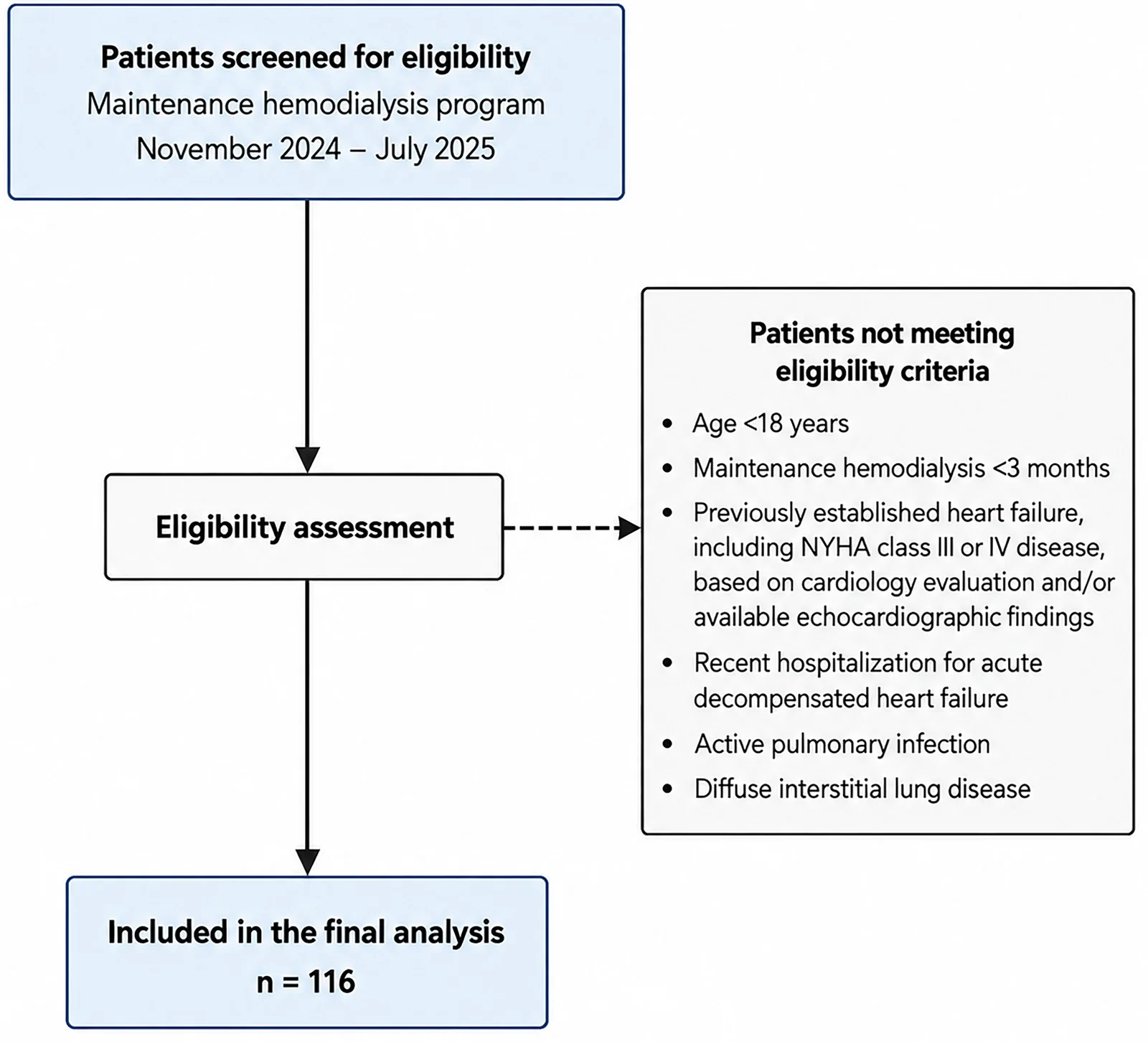
Flow diagram of patient selection and study procedures. Flow diagram showing the number of patients assessed for eligibility, reasons for exclusion, the final analytical cohort, and the standardized predialysis study procedures performed before the first hemodialysis session of the week.

**Table 1:** Baseline characteristics of the study population (n = 116).

Characteristic	Overall (n = 116)
Demographic characteristics	
Age, years	67 (56–74)
Male sex, n (%)	69 (59.5)
Time on hemodialysis, months	37 (16–68)
Vascular access	
Arteriovenous fistula, n (%)	22 (19.0)
Central venous catheter, n (%)	94 (81.0)
Predialysis systolic blood pressure, mmHg	147 (131–160)
Predialysis diastolic blood pressure, mmHg	80 (73–95)
Oxygen saturation, %	96 (96–97)
Interdialytic weight gain, kg	2.0 (1.0–3.1)
Interdialytic weight gain (% dry body weight)	3.3 (1.8–5.3)
BCM-derived overhydration, L	2.5 (1.3–3.6)
Residual urine output, mL/day	300 (200–500)
Hemoglobin, g/dL	12.0 (10.9–13.1)
Serum albumin, g/dL	4.0 (3.7–4.2)
Ferritin, ng/mL	830 (490–1170)
Transferrin saturation, %	24.2 (19.8–32.0)
LUS findings	
Total B-line count	8 (2–13)
Clinics	
Clinical congestion, n (%)	67 (57.8)
Clinical euvolemia, n (%)	49 (42.2)
LUS Pulmonary congestion (≥3 B-lines), n (%)	82 (70.7)
LUS Moderate-to-severe pulmonary congestion (≥10 B-lines), n (%)	48 (41.4)

Values are presented as median (interquartile range) or number (percentage), as appropriate. BCM, Body Composition Monitor.

Transthoracic echocardiography was available in 59 participants (50.9%) as part of routine clinical care. Median left ventricular ejection fraction was 56% (IQR, 53–61.5), and no participant had a clinical diagnosis of heart failure, consistent with the study exclusion criteria. Additional echocardiographic characteristics are presented in **Supplementary Table S2**.

### Clinical–ultrasound discordance and volume phenotypes

Among the 49 clinically euvolemic patients, 27 (55.1%) demonstrated ≥3 B-lines on LUS, fulfilling criteria for subclinical pulmonary congestion and illustrating substantial clinical–ultrasound discordance. This subgroup represented 23.3% of the overall study population.

Integration of clinical examination and LUS identified four distinct volume phenotypes. Concordant euvolemia was observed in 22 patients (19.0%), subclinical pulmonary congestion (clinical–ultrasound discordance) in 27 (23.3%), clinical congestion without ultrasound congestion in 12 (10.3%), and concordant clinical and ultrasound congestion in 55 (47.4%). Patients with concordant congestion demonstrated the highest interdialytic weight gain, BCM-derived overhydration, prevalence of intradialytic hypertension, and pulmonary B-line burden, whereas patients with concordant euvolemia consistently showed the lowest values for these parameters. Patients with subclinical pulmonary congestion exhibited intermediate BCM-derived overhydration despite the absence of clinical signs of congestion. Detailed characteristics of the four phenotypes are presented in **Table 2**, and their distribution is illustrated in **Figure 2**.

**Table 2:** Characteristics according to clinical–ultrasound volume phenotypes.

Characteristic	Concordant euvolemia (n = 22)	Clinical–ultrasound discordance (subclinical pulmonary congestion) (n = 27)	Clinical congestion without ultrasound congestion (n = 12)	Concordant congestion (n = 55)	P value
n (%)	22 (19.0)	27 (23.3)	12 (10.3)	55 (47.4)	—
Age, years	62 (48–68)	71 (60–78)	65 (54–69)	68 (56–74)	0.162
Male sex, n (%)	14 (63.6)	11 (40.7)	9 (75.0)	35 (63.6)	0.128
Time on hemodialysis, months	32 (17–66)	33 (13–54)	48 (18–59)	44 (16–76)	0.515
Interdialytic weight gain, kg	1.6 (0.3–2.3)	1.2 (0.5–2.0)	2.4 (1.5–4.0)	2.3 (1.5–3.6)	<0.001
Interdialytic weight gain, % dry body weight	2.4 (0.4–3.5)	2.0 (0.9–4.2)	3.3 (2.0–5.0)	3.8 (2.7–5.8)	0.002
BCM-derived overhydration, L	1.2 (0.1–2.4)	2.0 (1.2–3.2)	3.5 (2.2–4.0)	3.0 (1.8–4.4)	<0.001
Residual urine output, mL/day	400 (225–500)	400 (200–500)	350 (225–500)	300 (200–500)	0.815
Hemoglobin, g/dL	12.4 (11.4–13.5)	11.8 (10.5–13.4)	12.1 (10.9–13.2)	12.0 (10.5–12.8)	0.480
Albumin, g/dL	4.0 (3.8–4.3)	3.9 (3.6–4.2)	3.9 (3.7–4.2)	4.0 (3.7–4.2)	0.507
Ferritin, ng/mL	562 (412–945)	803 (336–1128)	722 (516–1181)	977 (540–1194)	0.150
Transferrin saturation, %	24.9 (20.2–33.1)	23.8 (19.5–29.7)	29.7 (19.9–36.9)	24.2 (20.3–32.1)	0.829
Intradialytic hypertension, n (%)	12 (54.5)	15 (55.6)	11 (91.7)	43 (78.2)	0.022
Total B-line count	1 (0–2)	8 (6–10)	0 (0–2)	13 (9–17)	<0.001

Values are presented as median (interquartile range) or number (percentage), as appropriate. P values compare the four clinical–ultrasound phenotypes. BCM, Body Composition Monitor.

**Figure 2:**
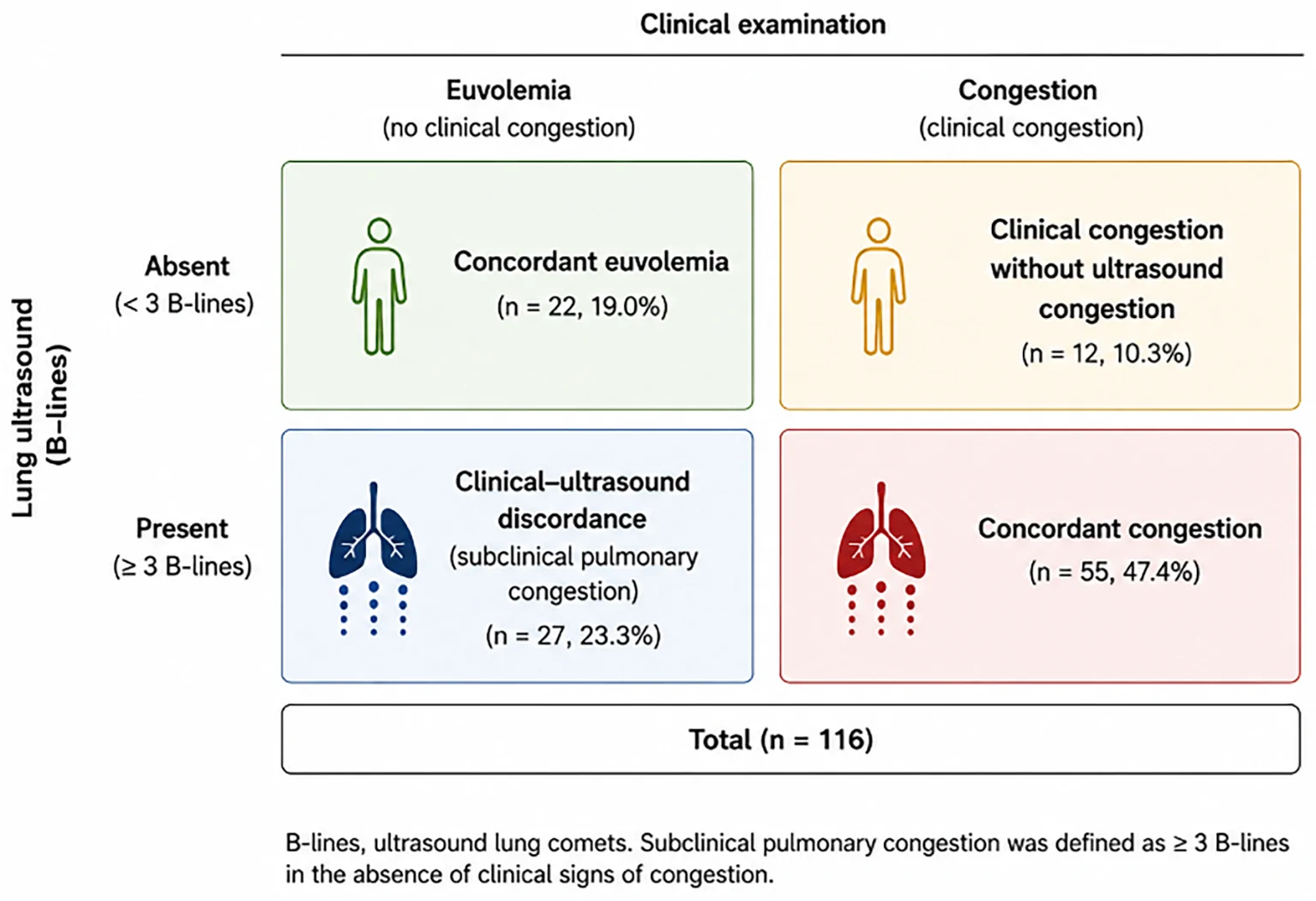
Clinical–ultrasound volume phenotypes. Distribution of the four phenotypes identified by combining standardized clinical examination with abbreviated six-zone lung ultrasound: concordant euvolemia, clinical congestion without ultrasound congestion, clinical–ultrasound discordance (subclinical pulmonary congestion), and concordant congestion.

### Relationship between LUS and conventional markers of volume status

Pulmonary B-line count demonstrated a weak positive correlation with BCM-derived overhydration (Spearman’s ρ = 0.26; P = 0.005). Interdialytic weight gain expressed in kilograms was not significantly correlated with pulmonary B-line burden (ρ = 0.16; P = 0.086). However, when normalized to dry body weight, a modest positive correlation was observed (ρ = 0.22; P = 0.017). Predialysis systolic blood pressure demonstrated a moderate positive correlation with BCM-derived overhydration (ρ = 0.40; P < 0.001), whereas no significant association was observed between systolic blood pressure and pulmonary B-line count. In contrast, predialysis diastolic blood pressure showed a weak but statistically significant positive correlation with pulmonary B-line burden (ρ = 0.23; P = 0.014). The relationship between BCM-derived overhydration and pulmonary B-line burden is illustrated in **Figure 3**.

**Figure 3:**
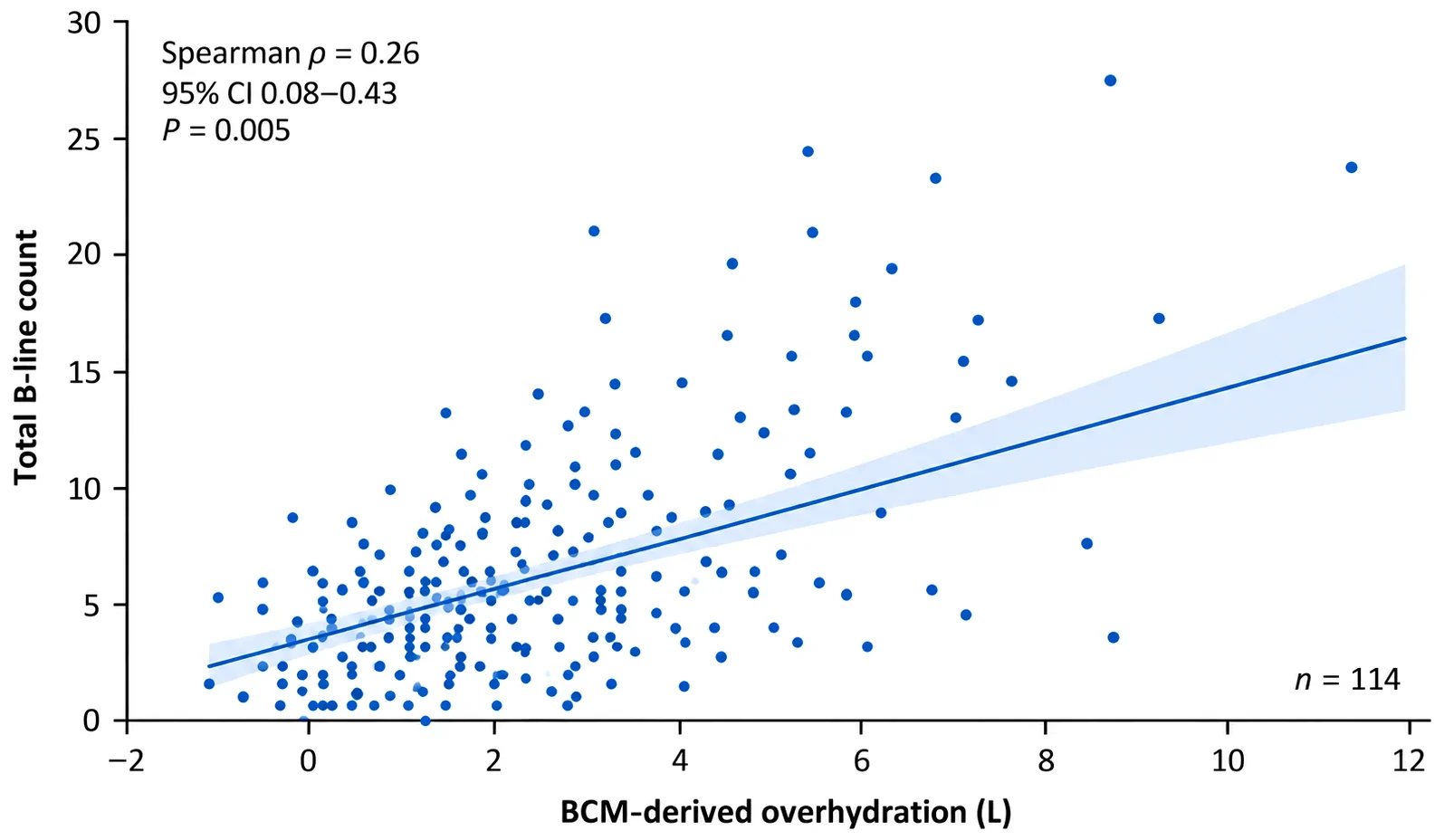
Relationship between BCM-derived overhydration and pulmonary B-line burden. Scatter plot showing the association between bioimpedance-derived overhydration and total pulmonary B-line count (Spearman’s ρ = 0.26; P = 0.005; n = 114).

### Agreement between volume assessment methods

Agreement among the bedside volume and congestion assessment methods was generally limited. Standardized clinical examination showed fair agreement with LUS when pulmonary congestion was defined as ≥3 B-lines (κ = 0.28; 95% CI, 0.11–0.45), improving to moderate agreement at the ≥10 B-line threshold (κ = 0.41; 95% CI, 0.26–0.57). Agreement between LUS and bioimpedance-defined overhydration was slight (κ = 0.16; 95% CI, −0.03 to 0.35), as was agreement between LUS and elevated interdialytic weight gain (κ = 0.08; 95% CI, −0.05 to 0.22). Complete agreement analyses are presented in **Table 3**.

**Table 3:** Agreement among bedside volume assessment methods.

Comparison	Cohen’s κ (95% CI)	Interpretation
Clinical examination vs LUS (≥3 B-lines)	0.28 (0.11–0.45)	Fair
Clinical examination vs LUS (≥10 B-lines)	0.41 (0.26–0.57)	Moderate
LUS (≥3 B-lines) vs BCM overhydration (>1.1 L)	0.16 (−0.03 to 0.35)	Slight
LUS (≥3 B-lines) vs IDWG >4% dry body weight	0.08 (−0.05 to 0.22)	Slight
Clinical examination vs BCM overhydration	0.20 (0.04–0.37)	Slight
Clinical examination vs IDWG >4% dry body weight	0.16 (0.00–0.32)	Slight

BCM, Body Composition Monitor; CI, confidence interval; IDWG, interdialytic weight gain; LUS, lung ultrasound.

### Factors associated with subclinical pulmonary congestion

Among clinically euvolemic patients with complete-case data (n = 47), multivariable Poisson regression showed that BCM-derived overhydration was independently associated with subclinical pulmonary congestion. Each additional liter of overhydration was associated with a 24% higher prevalence of subclinical pulmonary congestion. Male sex was independently associated with a lower prevalence of subclinical pulmonary congestion (adjusted PR, 0.55; 95% CI, 0.34–0.91; P = 0.019). Age, interdialytic weight gain, time on hemodialysis, and intradialytic hypertension were not independently associated with the outcome after multivariable adjustment. Complete regression analyses are presented in **Table 4**. Results of the sensitivity analysis using a negative binomial model for continuous pulmonary B-line count are provided in **Supplementary Table S3** and **Supplementary Figure S1**.

**Table 4:** Factors associated with subclinical pulmonary congestion.

Variable	No subclinical pulmonary congestion (n = 21)	Subclinical pulmonary congestion (n = 26)	Crude PR (95% CI)	P	Adjusted PR (95% CI)	P
BCM-derived overhydration, L	1.2 (0.1–2.4)	1.9 (1.2–3.2)	1.15 (0.97–1.36)	0.114	1.24 (1.02–1.50)	0.028
Sex						
Female	7 (30.4)	16 (69.6)	Reference	—	Reference	—
Male	14 (58.3)	10 (41.7)	0.60 (0.35–1.03)	0.065	0.55 (0.34–0.91)	0.019
Age, years	62.0 (48.0–68.0)	70.5 (59.5–76.8)	1.02 (0.99–1.04)	0.164	1.01 (0.98–1.03)	0.647
Interdialytic weight gain, kg	1.6 (0.3–2.3)	1.2 (0.5–2.0)	0.96 (0.77–1.20)	0.744	0.92 (0.72–1.18)	0.515
Time on hemodialysis, months	32 (17–67)	31 (13–49)	1.00 (0.99–1.00)	0.356	1.00 (0.99–1.00)	0.473
Intradialytic hypertension						
No	9 (45.0)	11 (55.0)	Reference	—	Reference	—
Yes	12 (44.4)	15 (55.6)	1.01 (0.60–1.70)	0.970	0.78 (0.46–1.31)	0.341

Values are presented as median (interquartile range) or n (row %). Prevalence ratios were estimated using robust Poisson regression. Adjusted analyses included all variables shown. Continuous variables are expressed per 1-unit increase. Two clinically euvolemic patients were excluded because of missing data. BCM, Body Composition Monitor; CI, confidence interval.

### Diagnostic performance of LUS for clinically overt congestion

Receiver operating characteristic analysis demonstrated that total pulmonary B-line count discriminated patients with clinically overt congestion with an area under the curve (AUC) of 0.74. According to the Youden index, approximately 9 B-lines represented the optimal threshold for discrimination. Using the prespecified cutoff of ≥10 B-lines, sensitivity was 60%, specificity was 84%, the positive predictive value was 83%, and the negative predictive value was 60%. The ROC curve is presented in **Supplementary Figure S2**.

## Discussion

The present study showed that clinically occult pulmonary congestion and discordance among clinical examination, BIS, and LUS are common in maintenance hemodialysis patients without clinically recognized heart failure. The most clinically relevant finding was that more than half of patients classified as clinically euvolemic by standardized clinical examination (27/49; 55.1%) had objective pulmonary congestion on abbreviated LUS, representing nearly one quarter (23.3%) of the overall cohort. Agreement between clinical examination and objective bedside methods was only modest, whereas bioimpedance-derived overhydration, but not interdialytic weight gain, remained independently associated with subclinical pulmonary congestion. To our knowledge, this is among the first studies from a Latin American maintenance hemodialysis population to comprehensively evaluate discordance among clinical examination, BIS, and abbreviated LUS in patients without clinically recognized heart failure. Performing all assessments on the same predialysis day using standardized protocols reduced the likelihood that the observed discordance reflected temporal variability between measurements.

The high prevalence of clinically occult pulmonary congestion observed in our cohort was consistent with previous reports. More than half of clinically euvolemic patients (55.1%) had ultrasound-defined pulmonary congestion despite the absence of dyspnea, peripheral edema, or abnormal pulmonary auscultation. Contemporary systematic reviews and meta-analyses consistently report pulmonary congestion in approximately one half to two thirds of maintenance hemodialysis patients despite differences in patient characteristics, ultrasound protocols, and B-line thresholds, supporting the concept that clinically silent pulmonary congestion is a reproducible finding across diverse dialysis populations (21, 24, 25). Likewise, Mallamaci et al. reported moderate-to-severe pulmonary congestion in approximately 63% of maintenance hemodialysis patients before dialysis, including 57% of asymptomatic individuals and nearly half of those considered clinically normohydrated (5).The Lung Water by Ultra-Sound Guided Treatment (LUST) study further demonstrated that conventional physical examination substantially underestimated pulmonary congestion detected by ultrasound, with pulmonary crackles showing a sensitivity of only approximately 22% for identifying significant pulmonary congestion (6). Collectively, these studies indicate that pulmonary congestion may remain clinically unapparent despite being detectable by ultrasound, highlighting the limited sensitivity of clinical examination alone for its detection in maintenance hemodialysis.

Several physiological considerations may help explain the observed discordance, although the present cross-sectional study cannot establish its underlying mechanism. Clinical examination detects the clinical manifestations of congestion only after cardiovascular and lymphatic compensatory mechanisms become insufficient, explaining its limited sensitivity for early pulmonary fluid accumulation (1, 2, 4, 6). BIS estimates systemic extracellular fluid expansion without directly quantifying pulmonary extravascular lung water, whereas interdialytic weight gain reflects net fluid accumulation between dialysis sessions but is influenced by sodium intake, residual kidney function, and the interdialytic interval rather than the physiological distribution of retained fluid (1, 2, 4, 18). In contrast, LUS quantifies pulmonary B-lines as a sonographic marker associated with increased extravascular lung water in the appropriate clinical context (9, 19). These differences provide a plausible physiological framework for understanding why the methods may classify the same patient differently, consistent with previous studies showing that different markers of volume status and pulmonary congestion do not necessarily behave in parallel (26, 27). Accordingly, the observed discordance is consistent with the hypothesis that these modalities capture partially distinct aspects of volume status and pulmonary congestion.

Another important finding was the consistently limited agreement among the most commonly used bedside methods for volume assessment, despite all assessments being performed immediately before the same midweek hemodialysis session. Clinical examination showed only fair agreement with LUS when pulmonary congestion was defined as ≥3 B-lines (κ = 0.28), improving to moderate agreement at the ≥10 B-line threshold (κ = 0.41). Agreement between LUS and either bioimpedance-defined overhydration (κ = 0.16) or elevated interdialytic weight gain (κ = 0.08) remained slight, demonstrating substantial classification discordance even when objective methods were compared under the same predialysis conditions. Chang et al. likewise demonstrated that physical examination, BIS, and LUS frequently classified the same patient differently despite same-day assessment (14). Similarly, Raz et al. reported limited agreement between conventional clinical assessment and LUS in clinically stable maintenance hemodialysis patients (16). Together, these findings suggest that limited agreement across bedside volume assessment methods has also been observed in other dialysis and chronic kidney disease populations.

Our multivariable analysis showed that bioimpedance-derived overhydration, but not interdialytic weight gain, was independently associated with subclinical pulmonary congestion. Specifically, each additional liter of overhydration measured by bioimpedance was associated with a 24% higher prevalence of ultrasound-defined pulmonary congestion, whereas interdialytic weight gain lost statistical significance after adjustment for potential confounders. These findings suggest that objective assessment of systemic extracellular fluid expansion provides information regarding pulmonary congestion that cannot be inferred from body weight changes alone. This interpretation is consistent with previous evidence. Ngoh et al. reported ultrasound-defined pulmonary congestion in 61% of patients considered normohydrated by bioimpedance, highlighting that systemic extracellular fluid expansion and pulmonary congestion frequently overlap only partially (28). Likewise, Kaysi et al. showed that pulmonary congestion and systemic extracellular fluid expansion followed partially independent physiological trajectories, with bioimpedance correlating with B-line burden only during one of the evaluated dialysis sessions (R² = 0.374; P = 0.007) (15). Previous physiological and interventional studies further demonstrated that pulmonary congestion may occur despite relatively modest systemic overhydration and that changes in pulmonary B-line burden during dialysis do not necessarily parallel changes in body weight or whole-body fluid status (29, 30). Together, these observations are consistent with incomplete overlap between systemic overhydration, interdialytic weight gain, and pulmonary congestion, although the present study cannot determine the mechanisms underlying these relationships.

From a clinical perspective, these findings support an integrated approach to bedside volume assessment rather than reliance on any single technique. Integrating BIS and LUS may provide additional information beyond that obtained from clinical examination or interdialytic weight gain alone, although the present study was not designed to determine whether such integration improves fluid management or clinical outcomes. This concept is supported by recent randomized and multimodal studies evaluating ultrasound-guided and combined bioimpedance–lung ultrasound strategies (31–33). Within this framework, discordance among bedside methods may be clinically informative and is consistent with the hypothesis that these techniques capture partially distinct aspects of volume status and pulmonary congestion. This evolving paradigm has been increasingly recognized as a major challenge and opportunity for contemporary nephrology (34).

Predialysis blood pressure also showed different relationships with systemic overhydration and pulmonary congestion. Systolic blood pressure correlated moderately with BCM-derived overhydration (ρ = 0.40; P < 0.001) but not with total pulmonary B-line count, whereas diastolic blood pressure showed only a weak correlation with total pulmonary B-line count (ρ = 0.23; P = 0.014). These findings are consistent with the multifactorial determinants of blood pressure in maintenance hemodialysis, including sodium balance, arterial stiffness, sympathetic activation, vascular remodeling, and extracellular fluid expansion (1, 2, 4).

The six-zone LUS protocol used in our study further supports the feasibility of multimodal bedside volume assessment in the hemodialysis setting. Previous validation studies have demonstrated that abbreviated LUS protocols maintain excellent diagnostic performance while substantially reducing examination time, thereby facilitating their incorporation into routine dialysis practice (10–12). Because LUS can be performed rapidly at the bedside without radiation exposure or contrast administration, its incorporation into the assessment of selected maintenance hemodialysis patients appears clinically feasible. Contemporary nephrology reviews further emphasize that POCUS is evolving from a complementary diagnostic technique to an integral component of bedside nephrology extending well beyond kidney imaging (35). Importantly, our findings should not be interpreted as advocating replacement of conventional clinical assessment but rather as supporting its integration with objective bedside techniques when volume status remains uncertain or clinically occult pulmonary congestion is suspected. These findings provide a rationale for future prospective studies evaluating whether multimodal volume assessment translates into more personalized fluid management and improved patient outcomes. Recent evidence from technology-guided strategies and lung ultrasound-guided clinical trials further supports this direction (13, 33).

### Strengths and limitations

This study has several important strengths. Volume status and pulmonary congestion were evaluated using an integrated bedside approach combining clinical examination, BIS, and an abbreviated six-zone LUS protocol during the same predialysis assessment, minimizing temporal variability and allowing direct comparison among the different assessment methods. A major methodological strength was the rigorous quality assurance applied to LUS assessment. All examinations were performed by two nephrologists formally trained in POCUS using a standardized acquisition protocol. Images were digitally stored, independently reviewed by both investigators, who remained blinded to clinical and bioimpedance findings, and disagreements were resolved by consensus before statistical analysis, minimizing observer-related bias and strengthening internal validity. An important strength also lies in the study’s conceptual framework. Rather than evaluating LUS as an isolated diagnostic or prognostic tool, we investigated discordance among the most commonly used bedside methods of volume assessment. Finally, our cohort contributes data from a Latin American maintenance hemodialysis population, a region that remains underrepresented in studies evaluating multimodal approaches to bedside volume assessment.

Several limitations should be acknowledged. First, the cross-sectional design precludes causal inference and does not permit evaluation of whether the observed discordance influences long-term cardiovascular outcomes or survival. Second, this was a single-center study, which may limit generalizability to other maintenance hemodialysis populations. The predominance of central venous catheter use reflects the local organization of vascular access care, as arteriovenous fistula creation requires referral to higher-complexity centers, which may limit generalizability. Third, transthoracic echocardiography was not systematically available in all participants. However, all patients underwent standardized cardiology evaluation as part of routine clinical care, and those with clinically recognized heart failure were excluded. Fourth, although all LUS examinations underwent independent duplicate review with consensus adjudication, formal interobserver agreement was not quantified. Finally, although the ≥3 B-line threshold used to define subclinical pulmonary congestion was prespecified before examination of the study data and informed by previous literature, no universally accepted threshold exists for subclinical pulmonary congestion using an abbreviated six-zone protocol. Sensitivity analyses using ≥5 and ≥10 B-lines yielded similar patterns.

## Conclusion

In maintenance hemodialysis patients without clinically recognized heart failure, physiological discordance among clinical examination, BIS and LUS was common, with abbreviated LUS identifying clinically occult pulmonary congestion in more than half of patients considered clinically euvolemic. These findings are consistent with the hypothesis that bedside methods capture partially distinct aspects of volume status and pulmonary congestion rather than interchangeable dimensions of a single construct. Accordingly, integrating clinical examination, BIS, and LUS may provide a more individualized interpretation of volume status and facilitate earlier recognition of pulmonary congestion than reliance on any single bedside technique, a potential clinical value that requires prospective evaluation. Future prospective studies should determine whether this multimodal approach translates into more personalized fluid management and improved cardiovascular and patient-centered outcomes.

## Data Availability

The original contributions presented in the study are included in the article/**Supplementary Material**, further inquiries can be directed to the corresponding author/s.
