# Supplementary material for: Physiological discordance between clinical examination, bioimpedance spectroscopy, and lung ultrasound in maintenance hemodialysis patients without heart failure
Source: Front Nephrol. 2026 Oct 9;6:2000700. doi: 10.3389/fneph.2026.2000700 (PMC13690536; doi:10.3389/fneph.2026.2000700)
Supplement: Supplementary file 1 [file Table1.docx]

Supplementary Material

# 1 Supplementary Figures and Tables

## 1.1 Supplementary tables

## Supplementary Table S1. Diagnostic performance of prespecified B-line thresholds for identifying clinically overt congestion.

| Threshold | Sensitivity, % | Specificity, % | PPV, % | NPV, % | Youden index | AUC (95% CI) |
| --- | --- | --- | --- | --- | --- | --- |
| ≥3 B-lines | 82.1 | 44.9 | 67.1 | 64.7 | 0.270 | 0.74 (0.64–0.83) |
| ≥5 B-lines | 74.6 | 53.1 | 68.5 | 60.5 | 0.277 | 0.74 (0.64–0.83) |
| ≥10 B-lines | 59.7 | 83.7 | 83.3 | 60.3 | 0.434 | 0.74 (0.64–0.83) |

**Abbreviations:** AUC, area under the receiver operating characteristic curve; NPV, negative predictive value; PPV, positive predictive value.

**Note:** Clinical congestion was the reference standard. The AUC was calculated from the continuous B-line count and therefore is identical across the prespecified dichotomization thresholds. The Youden index was calculated as sensitivity + specificity − 1.

## Supplementary Table S2. Echocardiographic characteristics of participants with available transthoracic echocardiography (n = 59)

| Variable | Value |
| --- | --- |
| Left ventricular ejection fraction, % | 56 (53–61.5) |
| TAPSE, mm | 20 (18–21) |
| No diastolic dysfunction | 9 (15.3%) |
| Grade I diastolic dysfunction | 38 (64.4%) |
| Grade II diastolic dysfunction | 12 (20.3%) |
| Low probability of pulmonary hypertension | 53 (89.8%) |
| Intermediate probability of pulmonary hypertension | 6 (10.2%) |
| Pericardial effusion, any | 4 (6.8%) |
| Mitral regurgitation, any | 39 (66.1%) |
| Tricuspid regurgitation, any | 35 (59.3%) |
| Aortic regurgitation, any | 24 (40.7%) |
| Aortic stenosis, any | 1 (1.7%) |
| Clinical diagnosis of heart failure | 0 (0.0%) |

*Values are presented as median (interquartile range) or n (%), as appropriate.* **Abbreviation:** TAPSE, tricuspid annular plane systolic excursion.

**Note:** Echocardiography was available in 59 of 116 participants (50.9%) as part of routine clinical care. No participant had a clinical diagnosis of heart failure, consistent with the study exclusion criteria.

## 1.2 Supplementary Figures

## Supplementary Figure 1. Factors associated with total pulmonary B-line count

Forest plot showing unadjusted and adjusted incidence rate ratios for total pulmonary B-line count. The adjusted negative binomial model included BCM-derived overhydration, interdialytic weight gain, residual diuresis, intradialytic hypertension, vascular access, dialysis frequency, dialysis-session duration, ferritin, and transferrin saturation. Squares represent adjusted IRRs and horizontal lines represent 95% confidence intervals. The vertical line indicates the null value (IRR = 1).

## Supplementary Figure 2. Diagnostic performance of total pulmonary B-line count for clinically overt congestion

Receiver operating characteristic curve for the continuous total B-line count using clinically overt congestion as the reference standard. The area under the curve was 0.74 (95% CI, 0.64–0.83). At the prespecified threshold of at least 10 B-lines, sensitivity was 59.7%, specificity was 83.7%, positive predictive value was 83.3%, and negative predictive value was 60.3%. The red point indicates the operating characteristics of the ≥10 B-line threshold.
